# African Swine Fever Virus K205R Induces ER Stress and Consequently Activates Autophagy and the NF-κB Signaling Pathway

**DOI:** 10.3390/v14020394

**Published:** 2022-02-15

**Authors:** Qi Wang, Luyu Zhou, Jiang Wang, Dan Su, Dahua Li, Yongkun Du, Guoyu Yang, Gaiping Zhang, Beibei Chu

**Affiliations:** 1 College of Veterinary Medicine, Henan Agricultural University, Zhengzhou 450046, China; wq18839830417@sina.com (Q.W.); zhouluyu12@sina.com (L.Z.); wangjiang@henau.edu.cn (J.W.); sudan98@sina.com (D.S.); lidahua52@sina.com (D.L.); duyongkun@henau.edu.cn (Y.D.); 2 Key Laboratory of Animal Biochemistry and Nutrition, Ministry of Agriculture and Rural Affairs, Zhengzhou 450046, China; yangguoyu@henau.edu.cn; 3 Key Laboratory of Animal Growth and Development, Henan Agricultural University, Zhengzhou 450046, China; 4 International Joint Research Center of National Animal Immunology, Henan Agricultural University, Zhengzhou 450046, China; 5 College of Animal Science & Techmology, Henan University of Animal Husbandry and Economy, Zhengzhou 450047, China

**Keywords:** ASFV K205R, stress granules, ER stress, autophagy, NF-κB

## Abstract

African swine fever virus (ASFV) is responsible for enormous economic losses in the global swine industry. The ASFV genome encodes approximate 160 proteins, most of whose functions remain largely unknown. In this study, we examined the roles of ASFV K205R in endoplasmic reticulum (ER) stress, autophagy, and inflammation. We observed that K205R was located in both the cytosolic and membrane fractions, and formed stress granules in cells. Furthermore, K205R triggered ER stress and activated the unfolded protein response through activating the transcription factor 6, ER to nucleus signaling 1, and eukaryotic translation initiation factor 2 alpha kinase 3 (EIF2AK3/PERK) signaling pathways. Moreover, K205R inhibited the serine/threonine kinase 1 and the mechanistic target of the rapamycin kinase signaling pathway, thereby activating unc-51 like autophagy activating kinase 1, and hence autophagy. In addition, K205R stimulated the translocation of P65 into the nucleus and the subsequent activation of the nuclear factor kappa B (NF-κB) signaling pathway. Inhibition of ER stress with a PERK inhibitor attenuated K205R-induced autophagy and NF-κB activation. Our data demonstrated a previously uncharacterized role of ASFV K205R in ER stress, autophagy, and the NF-κB signaling pathway.

## 1. Introduction

African swine fever virus (ASF), the only known DNA arbovirus, is prevalent in Africa, Europe, and Asia [1]. It causes a highly infectious and fatal hemorrhagic disease affecting the global swine industry [2]. Its genome contains approximately 160 major open reading frames (ORFs) and encoding products, including enzymes, structural proteins, and scaffolding proteins [3]. Some ASFV ORFs have been shown to be involved in regulating viral replication and host antiviral responses. For instance, ASFV A224L, A179L, EP153R, and DP71L inhibit apoptosis and prevent premature cell death, while ASFV E199L induces mitochondria-dependent apoptosis, supporting viral replication [4,5]. In addition, ASFV MGF-505-7R and MGF-505-11R negatively regulate the stimulator of IFN genes (STING)-dependent antiviral responses [6,7]. However, the function of most ASFV ORFs remains largely unknown. Given the lack of an effective vaccine against ASFV, it would be of great significance to characterize the function of ASFV proteins, providing new insight into ASFV vaccine development.

Various viruses induce endoplasmic reticulum (ER) stress and the subsequent unfolded protein response (UPR) [8]. Three signaling pathways, involving eukaryotic translation initiation factor 2 alpha kinase 3 (EIF2AK3/PERK), ER to nucleus signaling 1 (ERN1/IRE1), and activating transcription factor 6 (ATF6), are essential for the UPR [9]. ASFV structural protein p17 induces the generation of ER stress-reactive oxygen species, which inhibit cell proliferation [10]. ASFV activates the ATF6 branch of UPR, thus promoting infection [11]. CCAAT-enhancer-binding protein homologous protein (CHOP) is induced by ATF6 [12], but ASFV does not activate CHOP and instead inhibits the induction of CHOP through several exogenous stimuli [13]. Whether there are other ASFV proteins modulating ER stress remains unknown.

Autophagy is a conserved self-degradative process, which is important for cellular homeostasis and host-pathogen interactions [14]. ASFV E199L promotes autophagy through interacting with pyrroline-5-carboxylate reductase 2 and down-regulates its expression [15]. ASFV MGF505-11R interacts with the stimulator of interferon response cGAMP interactor 1 and stimulates its degradation through autophagy, suggesting that ASFV evades the innate immune response through hijacking autophagy [7]. In contrast, ASFV A179L has been found to inhibit autophagosome formation through interaction with Beclin-1 [16]. 

ASFV K205R has been found to be expressed in the early stages of infection, from 4 h post-infection, and is located in viral factories. Therefore, the K205R gene has received extensive research attention [16]. Adenovirus-vectored ASFV K205R elicits robust immune responses in swine [17]. ASFV K205R has high antigenicity and can be recognized by hyperimmune antisera from infected pigs, suggesting that K205R has the potential to be used for the detection of ASFV-specific antibodies [18]. In the present study, we report that ASFV K205R activates autophagy and the inflammatory response through triggering ER stress. Our results indicated a novel function of ASFV K205R.

## 2. Materials and Methods

### 2.1. Cells

The 3D4/21 (ATCC, CRL-2843), PK-15 (ATCC, CCL-33), and HeLa (ATCC, CCL-2) cells were cultured in DMEM (Gibco, Waltham, MA, USA) supplemented with 10% FBS (Gibco), 100 units/mL penicillin, and 100 mg/mL streptomycin sulfate (Sangon, Shanghai, China). All cells were grown in monolayers at 37 °C in 5% CO_2_. The 3D4/21 *P65*^−/−^, PK-15 *ATG5*^−/−^, and *Beclin-1*^−/−^ cells were used and cultivated as previously described [19,20].

### 2.2. Chemicals and Antibodies

GSK2606414 (S7307) was ordered from Selleck (Pittsburgh, PA, USA), and LPS (HY-D1056) was ordered from MedChemExpress (Monmouth Junction, NJ, USA). The antibodies, including anti-LC3 (12741), anti-LC3-Ⅱ (3868), anti-P62 (5114), anti-ATG5 (12994), anti-ATG12 (4180), anti-Beclin-1 (3495), anti-p-PERK (Thr980, 3179), anti-p-eIF2α (Ser51, 3398), anti-P65 (8242), anti-p-P65 (Ser536, 3033), anti-Lamin B1 (13435), anti-LAMP1 (9091), anti-IκBα (4814), anti-p-IκBα (Ser32, 2859), anti-mTOR (2983T), anti-p-mTOR (ser2448, 5536), anti-ULK1 (6439), anti-p-ULK1 (ser555, 5869), anti-p-ULK1 (ser757, 14202), anti-AKT (2920), and anti-p-AKT (Ser473, 4060) were ordered from Cell Signaling Technology (Danvers, MA, USA); anti-TOM20 (11802-1-AP), anti-GM130 (11308-1-AP), anti-Bip (11587-1-AP), anti-ATF6 (24169-1-AP), anti-PERK (24390-1-AP), anti-eIF2α (11170-1-AP), anti-ATF4 (10835-1-AP), anti-XBP1 (25997-1-AP), anti-β-actin (20536-1-AP), and anti-GFP (50430-2-AP) were ordered from Proteintech (Rosemont, IL, USA); anti-Calnexin (C4731) was ordered from Sigma-Aldrich (St. Louis, MO, USA); and anti-TIA-1 (sc-166247) was ordered from SCBT (Dallas, TX, USA).

### 2.3. Plasmids and Transfection

The coding sequence of ASFV K205R was synthesized by GenScript and cloned into pcDNA3.1 (Invitrogen, Waltham, MA, USA) fused with an HA tag sequence or into pEGFP-C1 (Clontech, Mountain View, CA, USA). All plasmids were transfected with Lipofectamine 3000 (Invitrogen), according to the manufacturer’s instructions.

### 2.4. Cell Viability Assays

The HeLa cells were seeded in 60-mm dishes at a density of 4 × 10^5^ cells per dish and transfected with pcDNA3.1 (vector, 5 μg) and K205R-HA plasmid (5 μg) for 24 and 48 h, respectively. The cell viability was determined with a CCK-8 cell counting assay kit (DingGuo, Beijing, China), according to the manufacturer’s instructions.

### 2.5. Immunoblotting Analysis

The cells were seeded in 60-mm dishes at a density of 7 × 10^5^ cells per dish and transfected with indicated plasmids (0–5 μg) for 24 h. The cells were collected in RIPA buffer (Solarbio, Beijing, China) supplemented with protease and phosphatase inhibitor cocktail (MedChemExpress). The protein concentrations of the lysates were quantified with a BCA Protein Assay Kit (DingGuo). The protein samples were separated by SDS-PAGE and transferred to membranes (Millipore, Billerica, MA, USA), which were incubated in 5% nonfat milk (Sangon) at room temperature for 1 h afterwards. The membranes were incubated with primary antibodies at 4 °C overnight and then incubated with horseradish-peroxidase-conjugated secondary antibodies (Jackson ImmunoResearch Laboratories, West Grove, PA, USA) for 1 h. The immunoblotting results were visualized with Luminata Crescendo Western HRP Substrate (Millipore) on a GE AI600 imaging system. The densitometric analysis of target proteins was performed with the ImageJ software (https://imagej.nih.gov/ij/ accessed on 27 September 2021).

### 2.6. Separation of the Soluble and Insoluble Fractions from Cells

The cells were seeded in 60-mm dishes at a density of 7 × 10^5^ cells per dish and transfected with indicated plasmids (5 μg) for 24 h. For separation of soluble and insoluble fractions, the cells were lysed in RIPA buffer (Solarbio) supplemented with protease and phosphatase inhibitor cocktail (MedChemExpress), and centrifuged at 12,000× *g* at 4 °C for 15 min. The supernatant was considered the soluble fraction, whereas the insoluble pellet was directly mixed with 1 × NuPage LDS Sample Buffer (Invitrogen) and heated at 100 °C for 30 min. The extracted fractions were subjected to immunoblotting analysis.

### 2.7. Nuclear and Cytoplasmic Extraction from Cells

The cells were seeded in 60-mm dishes at a density of 7 × 10^5^ cells per dish and transfected with indicated plasmids (5 μg) for 24 h. Nuclear and cytoplasmic extraction was performed with NEPER Nuclear and Cytoplasmic Extraction Reagents (Thermo Fisher Scientific, Waltham, MA, USA), according to the manufacturer’s instructions. The extracted fractions were subjected to immunoblotting analysis.

### 2.8. Separation of Cytosolic and Membrane Fractions from Cells

The cells were seeded in 60-mm dishes at a density of 7 × 10^5^ cells per dish and transfected with indicated plasmids (5 μg) for 24 h. Briefly, the cells were homogenized in 0.5 mL of homogenization buffer (10 mM HEPES pH 7.4, 10 mM KCl, 1.5 mM MgCl_2_, 5 mM sodium EDTA, 5 mM sodium EGTA, and 250 mM sucrose) supplemented with protease and phosphatase inhibitors (MedChemExpress), then centrifuged at 1000× *g* at 4 °C for 7 min. The pellet, containing the crude nuclear fraction, was discarded, and the supernatant was centrifuged at 12,000× *g* at 4 °C for 15 min. The resulting supernatant comprised the cytosol, and the pellet containing the membrane fraction was dissolved in lysis buffer (10 mM Tris HCl pH 6.8, 100 mM NaCl, 1% SDS, 1 mM EDTA, and 1 mM EGTA) supplemented with protease and phosphatase inhibitors. The whole cell lysate, cytosolic, and membrane samples were mixed individually with 4 × SDS loading buffer, boiled at 95 °C for 5 min, and subjected to SDS-PAGE and immunoblotting analysis.

### 2.9. The qRT-PCR

The cells were seeded in 12-well plates at a density of 3 × 10^5^ cells per dish and transfected with indicated plasmids (2 μg) for 24 h. Total RNA was isolated with TRIzol Reagent (TaKaRa, Shiga, Japan) and subjected to cDNA synthesis with a PrimeScript™ RT reagent Kit (TaKaRa). qRT-PCR was performed in triplicate with SYBR Premix Ex Taq (TaKaRa), according to the manufacturer’s instructions. The data were normalized to the level of *β-actin* expression in each individual sample. The melting curve analysis indicated formation of a single product in all cases. The 2^−ΔΔCt^ method was used to calculate relative expression changes. The primers used for qRT-PCR were as follows: *β-actin*-Fw: 5′-CTGAACCCCAAAGCCAACCGT-3′, *β-actin*-Rv: 5′-TTCTCCTTGATGTCCCGCACG-3′; *Atf4*-Fw: 5′-CCCTTTACGTTCTTGCAAACTC-3′, *Atf4*-Rv: 5′-GCTTCCTATCTCCTTCCGAGA-3′; *Gadd34*-Fw: 5′-AAGAGCCTGGAGAGAGGAGAG-3′, *Gadd34*-Rv: 5′-GTCCCCAGGTTTCCAAAAGCA-3′; *Chop*-Fw: 5′-CTCAGG AGGAAGAGGAGGAAG-3′, *Chop*-Rv: 5′-GCTAGCTGTGCCACTTTCCTT-3′; *Xbp1(s)*-Fw: 5′-GAGTCCGCAGCAGGTG-3′, *Xbp1(s)*-Rv: 5′-CCGTCAGAATCCATGGGG-3′; *Xbp1(t)*-Fw: 5′-TCCGCAGCACTCAGACTACGT-3′, *Xbp1(t)*-Rv: 5′-ATGCCCAAGAGGATATCAGACTC-3′; *ERdj4*-Fw: 5′-CAGAGAGATTGCAGAAGCATATGA-3′, *ERdj4*-Rv: 5′-GCTTCTTGGATCGAGTGTTTT-3′; *Il-6*-Fw: 5′-GCCTGAGGGCCATTCGGATA-3′, *Il-6*-Rv: 5′-TGTGCCCAGTGGACAGGTTT-3′; *Il-18*-Fw: 5′-AGGGACATCAAGCCGTGTTT-3′, *Il-18*-Rv: 5′-CGGTCTGAGGTGCATTATCTGA-3′; and *Tnfa*-Fw: 5′-CTGTAGGTTGCTCCCACCTG-3′, *Tnfa*-Rv: 5′-CCAGTAGGGCGGTTACAGAC-3′.

### 2.10. Immunofluorescence Analysis

The cells were seeded in 12-well plates with coverslips at a density of 3 × 10^5^ cells per dish and transfected with indicated plasmids (2 μg) for 24 h. The cells were fixed with 4% paraformaldehyde in PBS for 30 min at room temperature and were then washed three times with PBS. The cells were permeabilized in PBS containing 0.1% Triton X-100 and blocked with 10% FBS in PBS. The primary antibodies were diluted with 10% FBS in PBS and incubated with the cells for 1 h at room temperature. After being washed with PBS, the cells were incubated with Alexa Fluor 568 goat anti-mouse IgG or Alexa Fluor 568 goat anti-rabbit IgG (Invitrogen) for 1 h at room temperature. The cells were finally washed in PBS and mounted in ProLong Diamond with DAPI (Invitrogen). Images were captured with a Zeiss LSM 800 confocal microscope.

### 2.11. ELISA

The cells were seeded in 12-well plates with coverslips at a density of 3 × 10^5^ cells per dish and transfected with indicated plasmids (2 μg) for 24 h. Concentrations of porcine IL-18 were measured in the cell supernatants with ELISA kits (Advanced BioChemical, Lawrenceville, GA, USA), according to the manufacturer’s instructions.

### 2.12. Statistical Analysis

All data were obtained from three independent experiments for quantitative analyses and are expressed as means ± standard errors. All data were analyzed in Prism 7 software (GraphPad Software, Inc., San Diego, CA, USA) with two-tailed Student’s *t*-tests, and *p* < 0.05 was considered statistically significant. 

## 3. Results

### 3.1. Physical and Biochemical Parameters of ASFV K205R

**Figure 1:**
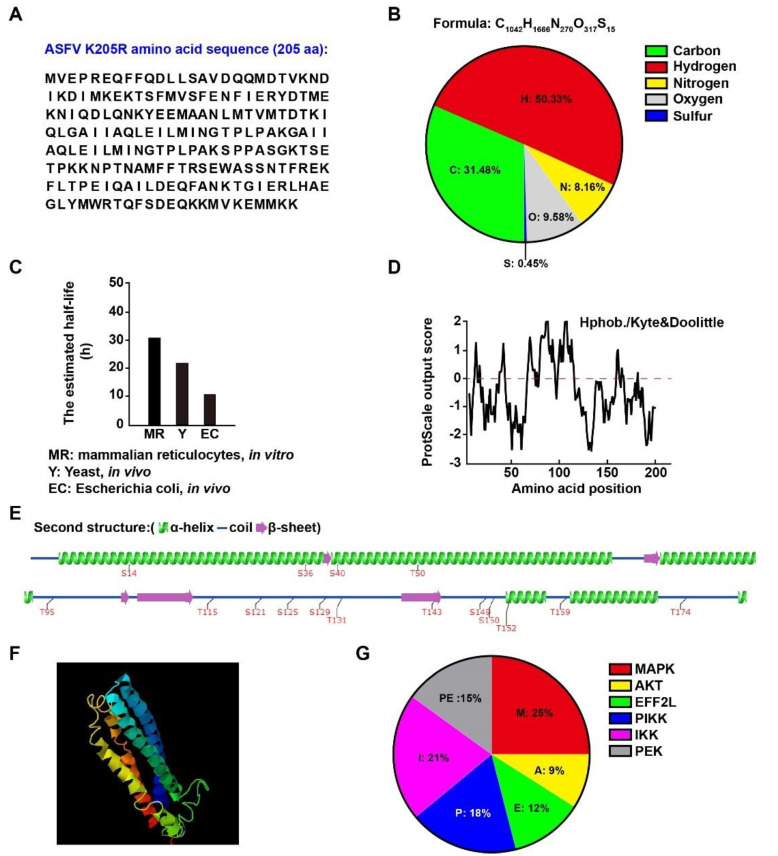
Physical and biochemical parameters of ASFV K205R. (**A**) Amino acid sequence of K205R. (**B**) Atomic composition of K205R. (**C**) Predicted half-life of K205R in mammalian reticulocytes, yeast and Escherichia coli. (**D**) Predicted hydropathicity and hydrophobicity of K205R. (**E**) Secondary structure and predicted phosphorylation sites of K205R. (**F**) Visualization of the predicted tertiary structure of K205R. (**G**) Distribution of phosphorylation sites in kinase families.

To preliminarily determine the function of ASFV K205R, we first analyzed its physical and biochemical parameters. K205R consisted of 205 amino acids comprising 31.48% carbon, 50.33% hydrogen, 9.58% oxygen, 8.16% nitrogen, and 0.45% sulfur (Figure 1A,B). We analyzed the half-life of K205R with ProtParam (https://web.expasy.org/protparam/ accessed on 12 November 2021). The estimated half-life of K205R in mammalian reticulocytes was 30 h, which was longer than the approximately 20 h in yeast and 10 h in *Escherichia coli* (Figure 1C). The hydrophobicity and hydrophilicity of K205R computed with ProtScale (https://web.expasy.org/protscale/ accessed on 12 November 2021) indicated that K205R was more hydrophobic than hydrophilic (Figure 1D). We also analyzed the secondary and tertiary structure of K205R with Group-based Prediction System Version 5.0 (http://gps.biocuckoo.cn/online_full.php accessed on 12 November 2021) and I-TASSER (https://zhanggroup.org/I-TASSER/ accessed on 12 November 2021). K205R consisted of four main α-helices and five β-sheets (Figure 1E,F). Sixteen potential phosphorylation sites in K205R were indicated by Group-based Prediction System Version 5.0, which might be phosphorylated by MAPK, AKT EFF2L, PIKK, IKK, and PEK kinases (Figure 1E,G).

### 3.2. Subcellular Localization of ASFV K205R

**Figure 2:**
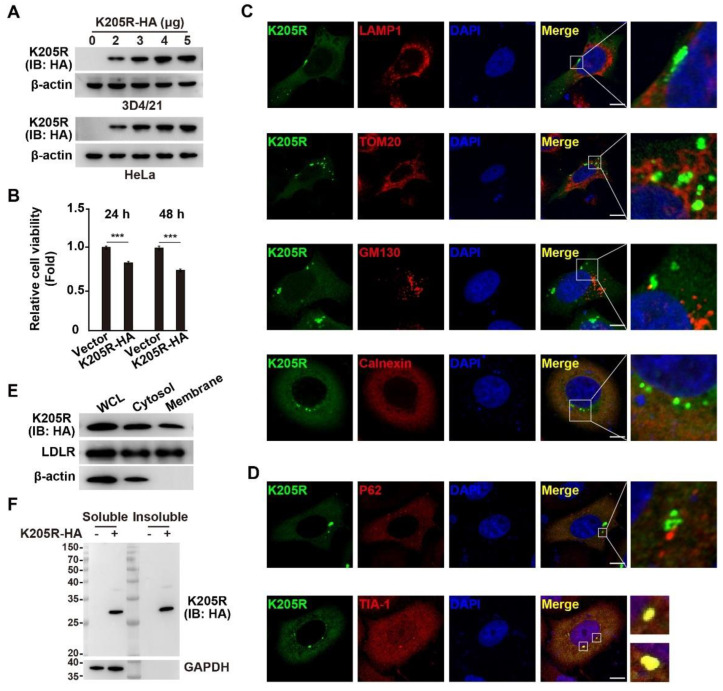
Expression and subcellular localization of K205R. (**A**) 3D4/21 and HeLa cells were transfected with K205R-HA plasmid as indicated for 24 h. The expression of K205R-HA was detected with immunoblotting analysis. (**B**) HeLa cells were transfected with empty vector or K205R-HA plasmid for 24 or 48 h. Cell viability was assessed with CCK-8 assays. *** *p* < 0.001. (**C**) HeLa cells were transfected with K205R-GFP plasmid for 24 h. Colocalization of K205R with LAMP1 (lysosome), TOM20 (mitochondria), GM130 (Golgi), and calnexin (ER) was analyzed with immunofluorescence analysis. Scale bar: 10 μm. (**D**) HeLa cells were transfected with K205R-GFP plasmid for 24 h. Colocalization of K205R with P62 (aggrephagy marker) and TIA-1 (SG marker) was determined with immunofluorescence analysis. Scale bar: 10 μm. (**E**) HeLa cells were transfected with K205R-HA plasmid for 24 h. The distribution of K205R in the cytosolic and membrane fractions was detected with immunoblotting analysis. (**F**) HeLa cells were transfected with empty vector or K205R-HA plasmid for 24 h. The distribution of K205R in soluble and insoluble fractions was detected with immunoblotting analysis.

To better understand K205R’s function, we constructed a plasmid for the expression of K205R fused with an HA tag. We transfected the plasmid into the 3D4/21 and HeLa cells and detected its expression by immunoblotting, which confirmed the expression of K205R-HA in both cell lines (Figure 2A). Furthermore, CCK-8 cell counting assay showed that K205R-HA resulted in lower cell viability than that of control cells (Figure 2B), indicating that K205R might affect cell viability. 

We then examined the subcellular distribution of K205R through co-localization assays with markers of lysosomes (indicated by LAMP1), mitochondria (indicated by TOM20), Golgi bodies (indicated by GM130), and ER (indicated by calnexin). K205R exhibited no clear co-localization with LAMP1, TOM20, and GM130, thus suggesting that K205R scarcely localized to lysosomes, mitochondria, and Golgi bodies (Figure 2C). K205R was diffusely distributed in the cells in a degree similar to calnexin (Figure 2C). Notably, K205R was found in punctate structures, in addition to areas with diffuse distribution (Figure 2C). Protein punctate structures may form as protein aggregates or stress granules (SGs) [21,22]. Protein aggregates interact with the selective autophagy receptor P62 and subsequently undergo aggrephagy [21]. We did not observe co-localization of K205R with P62, indicating that K205R did not form protein aggregates (Figure 2D). Interestingly, K205R clearly colocalized with the SG marker protein TIA-1 (Figure 2D). We further performed cell fractionation analysis to identify the localization of K205R in the cytosolic and membrane fractions. The low-density lipoprotein receptor is a cell surface receptor that is recycled in the cytosol after ligand binding [23]. K205R was distributed in both the cytosolic and membrane fractions, similar to the low-density lipoprotein receptor (Figure 2E). In addition, K205R was present in soluble and insoluble forms in the cells (Figure 2F). 

### 3.3. ASFV K205R Activates ER Stress

**Figure 3:**
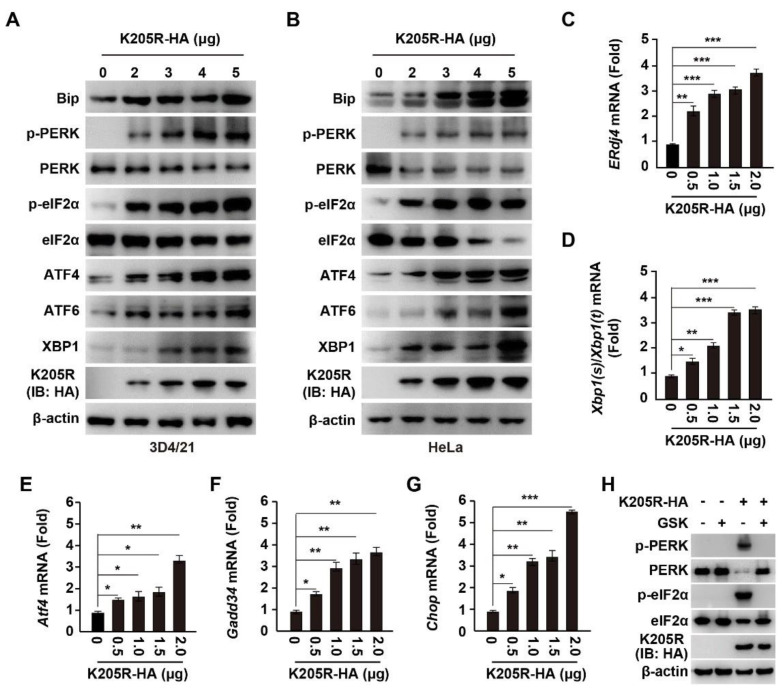
ASFV K205R induces ER stress. (**A**,**B**) 3D4/21 (**A**) and HeLa (**B**) cells were transfected with K205R-HA plasmid as indicated for 24 h. Bip, p-PERK, PERK, p-eIF2α, eIF2α, ATF4, ATF6, XBP1, K205R-HA, and β-actin were assessed with immunoblotting analysis. (**C**–**G**) 3D4/21 cells were transfected with K205R-HA plasmid as indicated for 24 h. The mRNA levels of *ERdj4* (**C**), *Xbp1(s)/Xbp1(t)* (**D**), *Atf4* (**E**), *Gadd34* (**F**), and *Chop* (**G**) were assessed with qRT-PCR analysis. * *p* < 0.05, ** *p* < 0.01, *** *p* < 0.001. (**H**) 3D4/21 cells were transfected with K205R-HA plasmid and treated with GSK2606414 (GSK, 10 μM) as indicated for 24 h. p-PERK, PERK, p-eIF2α, eIF2α, K205R-HA, and β-actin were assessed with immunoblotting analysis.

Cellular stress, such as ER stress, is a strong inducer of SG formation [24]. Therefore, we sought to determine whether K205R induces ER stress. There are three ER stress sensor pathways, IRE1, PERK and ATF6, which are critical to maintain ER homeostasis [25]. Using immunoblotting analysis, we detected that the expression of K205R in 3D4/21 cells stimulated ER stress in a K205R dose-dependent manner, as indicated by enhanced expression of ATF6 and phosphorylation of PERK, as well as the downstream effectors of ER stress, such as Bip, phosphorylated eIF2α, ATF4, and XBP1 (Figure 3A). This result suggested that K205R activated the IRE1, PERK, and ATF6 signaling pathways. We also obtained similar results in HeLa cells (Figure 3B). To further confirm the role of K205R in triggering ER stress, we performed qRT-PCR analysis to examine the transcript level of ER stress-responsive genes. The mRNA levels of *ERdj4*, processed *Xbp1* mRNA [*Xbp1(s)/Xbp1(t)*], *Atf4*, *Gadd34*, and *Chop* were all up-regulated in response to K205R expression (Figure 3C–G). Treatment of cells with a PERK inhibitor (GSK2606414, GSK) abolished K205R-induced phosphorylation of PERK and eIF2α (Figure 3H). Together, these results demonstrated that K205R induced ER stress.

### 3.4. ASFV K205R Activates Autophagy

**Figure 4:**
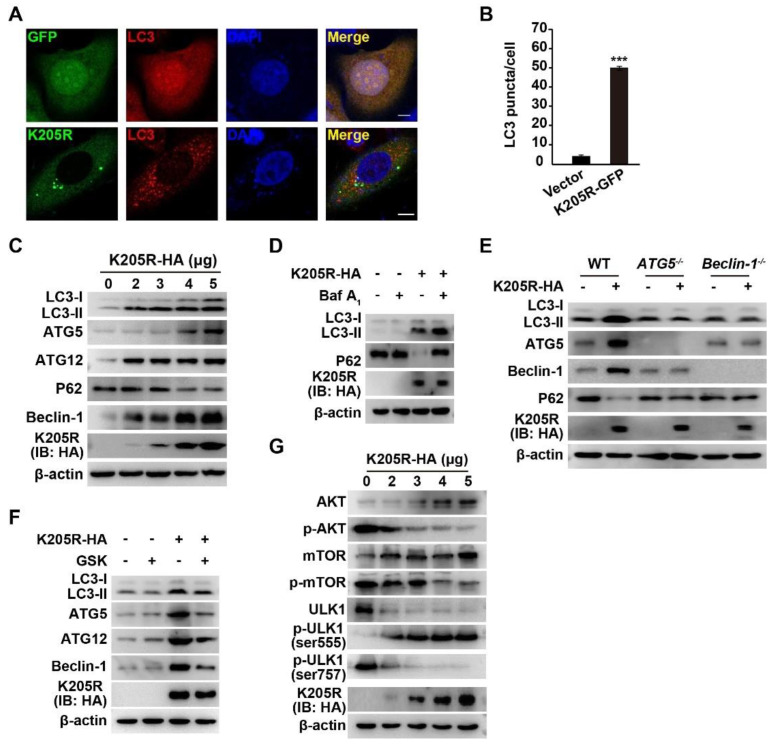
ASFV K205R activates autophagy. (**A**) HeLa cells were transfected with empty vector or K205R-GFP plasmid for 24 h. LC3 was monitored with immunofluorescence analysis. Scale bar: 10 μm. (**B**) Quantification of LC3 puncta per cell from A (*n* = 30). *** *p* < 0.001. (**C**) 3D4/21 cells were transfected with K205R-HA plasmid as indicated for 24 h. LC3-I, LC3-Ⅱ, ATG5, ATG12, P62, Beclin-1, K205R-HA, and β-actin were assessed with immunoblotting analysis. (**D**) 3D4/21 cells were transfected with K205R-HA plasmid and treated with bafilomycin A1 (10 μM) as indicated for 24 h. LC3-I, LC3-Ⅱ, ATG5, ATG12, P62, Beclin-1, K205R-HA, and β-actin were assessed with immunoblotting analysis. (**E**) PK15 WT, *ATG5*^−/−^, and *Beclin-1*^−/−^ cells were transfected with K205R-HA plasmid as indicated for 24 h. LC3-I, LC3-Ⅱ, ATG5, ATG12, P62, Beclin-1, K205R-HA, and β-actin were assessed with immunoblotting analysis. (**F**) 3D4/21 cells were transfected with K205R-HA plasmid and treated with GSK (10 μM) as indicated for 24 h. LC3-I, LC3-Ⅱ, ATG5, ATG12, Beclin-1, K205R-HA, and β-actin were assessed with immunoblotting analysis. (**G**) 3D4/21 cells were transfected with K205R-HA plasmid as indicated for 24 h. AKT, p-AKT, mTOR, p-mTOR, ULK1, p-ULK1 ser555, p-ULK1 ser757, K205R-HA, and β-actin were assessed with immunoblotting analysis.

We next attempted to examine whether K205R activates autophagy, given that ER stress is a potent trigger of autophagy [26]. We transfected K205R-GFP plasmids into the HeLa cells and then detected LC3 with immunofluorescence analysis. In the control cells, LC3 was spread throughout the cytosol and nucleus (Figure 4A). In the K205R-expressing cells, LC3 formed punctate structures, a characteristic of autophagosamal membrane formation (Figure 4A,B). We also examined K205R-induced autophagy by immunoblotting analysis. As shown in Figure 4C, increased expression of K205R resulted in enhanced expression levels of L3-II, ATG5, ATG12, and Beclin-1. When autophagy is activated, the selective autophagy receptor P62 is degraded in lysosomes and serves as an indicator of autophagic flux [21]. We observed that P62 expression decreased in response to K205R expression (Figure 4C). Bafilomycin A1, an inhibitor of the fusion of autophagosomes and lysosomes, can be used to analyze autophagic flux [27]. In the K205R expressing cells, bafilomycin A1 induced more LC3 accumulation than that in the control cells, but caused no P62 degradation (Figure 4D). 

Given that ATG5 and Beclin-1 are essential for the formation of autophagosomes [28], we verified the role of K205R in autophagy induction in *ATG5*^−/−^ and *Beclin-1*^−/−^ cells. K205R failed to induce autophagy in the *ATG5*^−/−^ and *Beclin-1*^−/−^ cells, as indicated by immunoblotting analysis of LC3-II, ATG5, ATG12, and Beclin-1 (Figure 4E). Inhibition of PERK by GSK in the K205R-transfected cells resulted in lower expression of LC3-II, ATG5, ATG12, and Beclin-1 than that in the K205R-transfected cells, further suggesting that K205R activated autophagy through ER stress (Figure 4F). The AKT/mTOR pathway negatively regulates unc-51 like autophagy activating kinase 1 (ULK1), and hence autophagy [29]. Therefore, we attempted to determine whether the AKT/mTOR/ULK1 signaling pathway is involved in K205R-induced autophagy. We observed that phosphorylated AKT and mTOR decreased when cells expressed K205R (Figure 4G). This result indicated that the AKT/mTOR pathway was inhibited by K205R. Phosphorylation of ULK1 Ser555 was enhanced, whereas phosphorylation of ULK1 Ser757 was decreased in response to K205R expression, suggesting that K205R activated ULK1 (Figure 4G). Collectively, these data indicated that K205R activated autophagy through the AKT/mTOR/ULK1 signaling pathway.

### 3.5. ASFV K205R Activates the NF-κB Signaling Pathway

**Figure 5:**
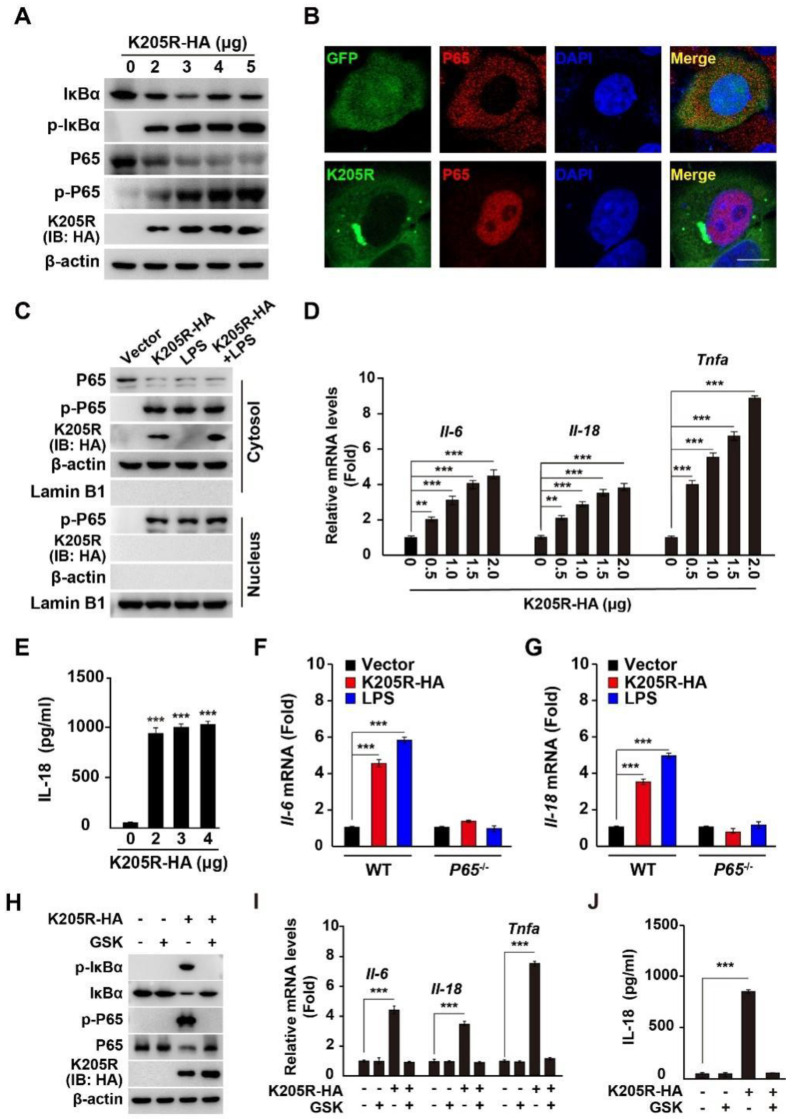
ASFV K205R activates the NF-κB signaling pathway. (**A**) 3D4/21 cells were transfected with K205R-HA plasmid as indicated for 24 h. IκBα, p-IκBα, P65, p-P65, K205R-HA, and β-actin were assessed with immunoblotting analysis. (**B**) HeLa cells were transfected with empty vector or K205R-GFP plasmid for 24 h. The translocation of P65 into the nucleus was assessed with immunofluorescence analysis. Scale bar: 10 μm. (**C**) HeLa cells were transfected with K205R-HA and treated with LPS (1 mg/mL) as indicated for 24 h. P65 and p-P65 in the cytosol (indicated by β-actin) and nucleus (indicated by Lamin B1) were assessed with immunofluorescence analysis. (**D**) 3D4/21 cells were transfected with K205R-HA plasmid as indicated for 24 h. The mRNA levels of *Il-6*, *Il-18*, and *Tnfa* were assessed with qRT-PCR analysis. ** *p* < 0.01, *** *p* < 0.001. (**E**) 3D4/21 cells were transfected with K205R-HA plasmid as indicated for 24 h. IL-18 in the medium was quantified with ELISA. *** *p* < 0.001. (**F**,**G**) 3D4/21 WT and *P65*^−/−^ cells were transfected with K205R-HA and treated with LPS (1 mg/mL) as indicated for 24 h. The mRNA levels of *Il-6* (**F**) and *Il-18* (**G**) were assessed with qRT-PCR analysis. *** *p* < 0.001. (**H**) 3D4/21 cells were transfected with K205R-HA plasmid and treated with GSK (10 μM) as indicated for 24 h. p-IκBα, IκBα, p-P65, P65, K205R-HA, and β-actin were assessed with immunoblotting analysis. (**I**) 3D4/21 cells were transfected with K205R-HA plasmid and treated with GSK (10 μM) as indicated for 24 h. The mRNA levels of *Il-6*, *Il-18*, and *Tnfa* were assessed with qRT-PCR analysis. *** *p* < 0.001. (**J**) 3D4/21 cells were transfected with K205R-HA plasmid and treated with GSK (10 μM) as indicated for 24 h. IL-18 in the medium was quantified with ELISA. *** *p* < 0.001.

It is known that ER stress can elicit proinflammation [25], therefore we sought to determine whether K205R activates the NF-κB signaling pathway. Phosphorylation of the IκBα and P65 subunits of nuclear factor kappa B (NF-κB) is essential for P65 translocation into the nucleus and subsequent NF-κB activation [30]. We observed that K205R expression resulted in the phosphorylation of IκBα and P65, as indicated by immunoblotting analysis (Figure 5A). Immunofluorescence analysis suggested that P65 was translocated into the nucleus in the presence of K205R expression (Figure 5B). We further analyzed NF-κB activation by cell fractionation analysis of P65 with β-actin as a cytosolic marker and Lamin B1 as a nuclear marker. As shown in Figure 5C, LPS (a well-known NF-κB activator) stimulated P65’s phosphorylation and translocation into the nucleus (Figure 5C). Expression of K205R-HA with simultaneous treatment of cells with LPS (K205R-HA + LPS) promoted the translocation of phosphorylated P65 into the nucleus (Figure 5C). 

Given that NF-κB activation triggers the expression of proinflammatory cytokines, we next examined the transcription of *Il-6*, *Il-18*, and *Tnfa* by qRT-PCR analysis. K205R increased the mRNA levels of *Il-6*, *Il-18*, and *Tnfa* in a K205R dose-dependent manner (Figure 5D). K205R stimulated IL-18 secretion into the culture medium, as indicated by ELISA analysis (Figure 5E). We further verified K205R-induced NF-κB activation in 3D4/21 *P65*^−/−^ cells. Neither LPS nor K205R stimulated the transcription of Il-6 and Il-18 in the 3D4/21 *P65*^−/−^ cells (Figure 5F,G). We finally determined whether inhibition of ER stress by a PERK inhibitor might abrogate K205R-mediated activation of the NF-κB signaling pathway. In the K205R expressing cells, GSK treatment prevented the phosphorylation of IκBα and P65 (Figure 5H). GSK treatment also inhibited the transcription of *Il-6*, *Il-18*, and *Tnfa*, as well as the secretion of IL-18 when K205R-HA was expressed in cells (Figure 5I,J). Together, these results demonstrated that K205R activated the NF-κB signaling pathway.

## 4. Discussion

ASFV causes an acute and fatal disease affecting domestic pigs. Its genome encodes more than 160 proteins. Better understanding of the roles of ASFV proteins would be valuable in supporting vaccine development, given that no vaccine is available for the prevention and control of ASFV. In the present study, we examined the roles of ASFV K205R in ER stress, autophagy, and inflammation. K205R induced ER stress, thereby activating the UPR via the ATF6, IRE1, and PERK signaling pathways. Subsequently, K205R stimulated autophagy and NF-κB activation, which were prevented by inhibition of ER stress. Our results suggested that K205R induced ER stress and consequently activated autophagy and the NF-κB signaling pathway.

K205R has been shown to have high antigenicity and can be used for the detection of ASFV-specific antibodies [18]. Adenovirus-vectored K205R has been found to elicit robust immune responses in swine, suggesting that K205R may be an effective component of a prototype vaccine [17]. K205R is diffusely distributed throughout cells, and is found in viral factories [31]. We observed that K205R formed SG in cells. Moreover, K205R was localized in the cytosolic and membrane fractions. Although K205R contained four main α-helices, it might not be a transmembrane protein. SG are membraneless ribonucleoprotein-based cellular compartments associated with the ER [32,33]. Therefore, we speculated that K205R might form SG tethered to the ER, thereby resulting in the observed membrane distribution of K205R.

Several lines of evidence indicate that ASFV modulated ER stress. Xia and colleagues have shown that ASFV P17 inhibited cell proliferation through ER stress and ROS-mediated cell cycle arrest [10]. Our data demonstrated that K205R decreased cell viability; therefore, K205 might promote either cell death or cell cycle arrest. Whether K205R induces ROS requires further investigation. ASFV induces the ATF6 branch of the UPR, but not the PERK pathways, which promote ASFV infection [11]. In contrast, ASFV does not activate ATF6-regulated CHOP and instead inhibits the induction of CHOP/GADD153 via several exogenous stimuli [13]. Our data indicated that K205R activates the IRE1, PERK, and ATF6 signaling pathways. Other ASFV proteins might possibly modulate ER stress. The role of ASFV in modulating ER stress depends on the synergistic effects of ASFV proteins that participate in ER stress. We acknowledge that more data is needed to confirm that K205R induces ER stress under physiological conditions.

Innate immunity is the front-line defense against viral infections [34]. Cyclic guanosine monophosphate/adenosine monophosphate synthase (cGAS) and STING are crucial innate immune proteins involved in cytosolic DNA sensing [35]. Although ASFV controls interferon beta production through the cGAS-STING pathway, this virus exploits autophagy to interfere with this pathway [36]. ASFV MGF-505-7R negatively regulates the cGAS-STING-mediated signaling pathway through autophagy-mediated degradation of STING [6]. Moreover, ASFV MGF505-11R promotes STING degradation by autophagy for negative regulation of the cGAS-STING signaling pathway [7]. ASFV E199L promotes cell autophagy through the interaction of PYCR2 [15]. We found that K205R activated autophagy through ER stress. The roles of ASFV E199L-induced and K205R-induced autophagy in STING degradation and innate immune evasion are worthy of further study. Notably, we found that K205R activated the NF-κB signaling pathway, thereby protecting the host against ASFV infection. This understanding of the complicated roles of K205R in cellular responses provides new insight into virus–host interactions.

## Data Availability

All available data are presented in the article.
